# Fetal circulating human resistin increases in diabetes during pregnancy and impairs placental mitochondrial biogenesis

**DOI:** 10.1186/s10020-020-00205-y

**Published:** 2020-08-06

**Authors:** Shaoning Jiang, April M. Teague, Jeanie B. Tryggestad, Timothy J. Lyons, Steven D. Chernausek

**Affiliations:** 1 Department of Pediatrics, Section of Diabetes and Endocrinology, Harold Hamm Diabetes Center, University of Oklahoma Health Sciences Center, 1200 Children’s Ave Suite 4500, Oklahoma City, OK 73104 USA; 2 Division of Endocrinology, Diabetes, and Metabolic Diseases at the Medical University of South Carolina, Charleston, SC USA

**Keywords:** Human resistin, Diabetes during pregnancy, Placenta, Mitochondria

## Abstract

**Background:**

Diabetes during pregnancy affects placental mitochondrial content and function, which has the potential to impact fetal development and the long-term health of offspring. Resistin is a peptide hormone originally discovered in mice as an adipocyte-derived factor that induced insulin resistance. In humans, resistin is primarily secreted by monocytes or macrophages. The regulation and roles of human resistin in diabetes during pregnancy remain unclear.

**Methods:**

Fetal resistin levels were measured in cord blood from pregnancies with (*n* = 42) and without maternal diabetes (*n* = 81). Secretion of resistin from cord blood mononuclear cells (CBMCs) was measured. The actions of human resistin in mitochondrial biogenesis were determined in placental trophoblastic cells (BeWo cells) or human placental explant.

**Results:**

Concentrations of human resistin in cord sera were higher in diabetic pregnancies (67 ng/ml) compared to healthy controls (50 ng/ml, *P* < 0.05), and correlated (*r* = 0.4, *P* = 0.002) with a measure of maternal glycemia (glucose concentration 2 h post challenge). Resistin mRNA was most abundant in cord blood mononuclear cells (CBMCs) compared with placenta and mesenchymal stem cells (MSCs). Secretion of resistin from cultured CBMCs was increased in response to high glucose (25 mM). Exposing BeWo cells or human placental explant to resistin decreased expression of peroxisome proliferator-activated receptor gamma coactivator 1-alpha (PGC-1α), mitochondrial abundance, and ATP production.

**Conclusions:**

Resistin is increased in fetal circulation of infants exposed to the diabetic milieu, potentially reflecting a response of monocytes/macrophages to hyperglycemia and metabolic stresses associated with diabetes during pregnancy. Increased exposure to resistin may contribute to mitochondrial dysfunction and aberrant energy metabolism characteristic of offspring exposed to diabetes in utero.

## Background

Diabetes during pregnancy, including pre-gestational diabetes and gestational diabetes (GDM), affects fetal growth, which is linked to the development of obesity, diabetes, and cardiovascular diseases in later life (Fetita et al. 2006; Dabelea and Crume 2011; Damm et al. 2016; Friedman 2018). Approximately 16% of pregnant women globally have diabetes during pregnancy (International Diabetes Federation, Diabetes Atlas 9th edition, 2019), and the percentage continues to increase, contributing significantly to the increased prevalence of diabetes and obesity in subsequent generations. The placenta plays a key role in fetal growth and development by supplying nutrients and oxygen. Diabetes during pregnancy alters placental structure and function with aberrant vascularization, increased inflammation, and impaired energy metabolism (Jarmuzek et al. 2015; Osmond et al. 2000; Muralimanoharan et al. 2016). As the interface between maternal and fetal circulation systems, the placenta can be affected by changes in both maternal and fetal circulating factors in response to the diabetic milieu (Desoye and Hauguel-de Mouzon 2007).

Resistin is a secreted protein implicated in the pathogenesis of obesity and type 2 diabetes. It was discovered in rodents as an adipocyte-derived factor which induces insulin resistance (Steppan et al. 2001). Human and murine resistin only share 59% homology at the amino acid level (Ghosh et al. 2003). Unlike rodent resistin, human resistin is predominantly produced by peripheral blood mononuclear cells (PBMCs), macrophages, and bone marrow cells (Schwartz and Lazar 2011). Human resistin has been shown to induce expression of proinflammatory cytokines and adhesion molecules in the settings of inflammation and endothelial dysfunction. Given the strong relationship between inflammation and metabolism, there is mounting evidence suggesting a role for human resistin in the pathological processes of metabolic diseases, including obesity, diabetes, and cardiovascular diseases (Lazar 2007; McTernan et al. 2002). However, the precise mechanism by which resistin impacts these processes has not been clearly defined as several studies have failed to identify an association of resistin levels with obesity or type 2 diabetes (Gerber et al. 2005; Pfutzner et al. 2003). Resistin has been implicated in the insulin resistance observed in normal pregnancy, as the level of resistin increases with gestational age and decreases after delivery (Chen et al. 2005). Conflicting evidence exists regarding the association of maternal resistin levels with GDM. Recent meta-analysis suggests GDM is associated with increased maternal resistin levels (Hu et al. 2019), while resutls of another meta-analysis do not indicate a significant change of resitin levels in gestational diabetes (Bellos et al. 2019). Available prospective data are also inconsistent regarding the link of maternal resistin to the later development of GDM (Bao et al. 2015). Much less is known about fetal resistin levels and current studies examining the fetal levels of resistin in diabetes during pregnancy are inconsistent (Shang et al. 2018; Oncul et al. 2013; Mohamed et al. 2010). The aims of the present study are to assess the regulation and function of human resistin in fetal circulation and how it affects placenta in diabetes during pregnancy.

Our previous studies demonstrate that maternal diabetes is associated with decreased PGC-1α/TFAM/mitochondrial biogenesis signaling in human placenta (Jiang et al. 2017). The present studies identify resistin as a potential mediator of that phenomenon by demonstrating increases in resistin concentration in the fetal circulation in pregnancies complicated by diabetes and inhibition of mitochondrial biogenesis and metabolism by resistin in the placenta. The production of resistin by fetal mononuclear cells reported here provides evidence for resistin as a link between the inflammatory response and energy metabolism in diabetes during pregnancy.

## Methods

### Subjects for cord serum samples

Pregnant Native American or Hispanic women with diabetes (*N* = 42, including 31 gestational diabetes and 11 pre-gestational type 2 diabetes), or non-diabetic controls (*N* = 81) were enrolled into a prospective longitudinal study on the impact of in utero exposure to DM, as previously described (Teague et al. 2015). Gestational or type 2 diabetes was diagnosed according to ADA guidelines (American Diabetes A 2003). Women with type 2 diabetes were defined as those diagnosed before pregnancy. Women were excluded if they delivered prior to 37 weeks gestation, had type 1 diabetes, pre-eclampsia, chronic hypertension, renal disorders or a smoking history during pregnancy. They were also excluded if the infants were small for gestational age, had a major malformation, or chromosome abnormality. Maternal glucose concentrations measured 2 h after oral glucose challenge (OGTT, Fig. 2) during the second trimester of pregnancy were obtained from clinical records. Cord blood and maternal blood (if available) were obtained after delivery, and cord and maternal serum resistin levels were measured. The protocol was approved by the Institutional Review Boards of the University of Oklahoma Health Science Center, the Chickasaw Nation, and the Choctaw Nation of Oklahoma. The samples collected under this protocol were not used for isolating cord blood mononuclear cells, mesenchymal stem cells, or placental explant culture.

### Studies using human cord blood mononuclear cells (CBMCs), mesenchymal stem cells (MSCs) and placental explant culture

CBMCs, MSCs, and placental explants were isolated respectively from cord blood, cord tissue, or placenta obtained at term from healthy human subjects recruited in a separate study cohort as described previously (Jiang et al. 2020). The protocol was approved by the Institutional Review Board of the University of Oklahoma Health Science Center. CBMCs were isolated from cord blood of non-diabetic healthy individuals by Ficoll density gradient centrifugation. The cord blood was diluted 1: 3 in PBS (without Ca^2+^ and Mg^2+^), layered over Ficoll buffer, and centrifuged at 400 g for 35 min. The interphase cell layer was collected and washed in PBS for 3 times. The CBMCs were plated and cultured in Dulbecco’s Modified Eagle Medium with 10% Fetal Bovine Serum followed by treatment with TNFα (100 ng/ml), high glucose (25 mM), palmitate acid (0.6 mM), or 4-hydroxynonenal (4-HNE, 0.6 mM) for 16 h. Mesenchymal stem cells (MSCs) were isolated from Wharton’s Jelly of cord tissue as previously described (Boyle et al. 2016). For Placental explant culture, two pieces of placental tissue were collected from healthy subjects within 15 min after delivery, stripped of connective tissues, and dissected to small pieces (about 2 mm). The placental villous explants were cultured in 6-well plate at 37 °C in 5% CO_2_ in Ham’s F-12 medium (Gibco/Life Technologies, Grand Island, NY) supplemented with 10% FBS (Mediatech, Manassas, VA), 100 μM MEM Non-Essential Amino Acids (Gibco/Life Technologies, Grand Island, NY), and 0.5% penicillin/streptomycin/amphotericin B (Gibco/Life Technologies, Grand Island, NY) and were treated with indicated doses of resistin or vehicle for 24 h in culture.

### ELISA

The concentrations of resistin in serum and cell culture media were measured using human Resistin DuoSet ELISA kit (R&D Systems, Minneapolis, MN) according to manufacturer’s protocol. Briefly, ELISA plates were coated with capture antibody overnight at room temperature followed by blocking with Reagent Diluent (DuoSet ELISA Reagent Kit) for 2 h. One hundred microliters cell culture media, or diluted fetal or maternal serum (1:40 in PBS), along with serial-diluted standards (0–4 ng/ml) were loaded to the plates and incubated overnight at 4 °C, followed by adding detection antibody and streptavidin-HRP subsequently. Optical density was determined using a microplate reader at 450 nm. The detection range of the assay is 0.0625 ng/ml to 4 ng/ml with intraplate coefficient of variation of the duplicates less than 10% and inter-plate coefficient of variation less than 15%.

### RNA extraction

Total RNA was extracted from BeWo cells (a human placental trophoblast cell line derived from a choriocarcinoma) using commercially available kits (miRNeasy, Qiagen, Valencia, CA) according to the manufacturer’s instructions. Isolated total RNA was quantified by a NanoDrop ND-1000 spectrophotometer (Thermo Scientific, Wilmington, DE).

### qPCR analysis

Reverse transcription (RT) was done with SuperScript VILO cDNA Synthesis Kit according to the manufacturer’s instructions (Invitrogen). Quantitative real-time PCR was performed using TaqMan Real-Time PCR Probes for PGC-1α or GAPDH (Life Technologies). Results were calculated using the 2^−ΔΔCt^ method normalized to endogenous control GAPDH.

### Western blot analysis

Western blot analysis was performed as described previously (Jiang et al. 2017). Placental explant samples or BeWo cells were lysed and homogenized in protein lysis buffer containing a protease and phosphatase inhibitor cocktail (Pierce Biotechnology, Rockford, IL). Protein concentrations were measured by BCA assay (Pierce, Rockford, IL). Thirty μg of protein lysate was reduced in laemmli sample buffer with dithiothreitol, and subjected to sodium dodecyl sulfated polyacrylamide gel electrophoresis (SDS-PAGE), then transferred to polyvinylidene fluoride (PVDF) membrane and incubated with antibodies specific for PGC-1α, PDH, or β-actin (Cell Signaling Technology, Danvers, MA). The proteins of interest were detected by enhanced chemiluminescence (Pierce, Rockford, IL) and analyzed by imaging densitometry with Image Lab Software (Bio-Rad, Hercules, CA).

### Mitochondrial DNA copy number

DNA was isolated from placental tissue using the GenElute Mammalian Genomic DNA Miniprep Kit (Sigma, St. Louis, MO) with proteinase K and RNase treatment, according to the manufacturer’s instructions. Mitochondrial DNA copy number was estimated by comparing the abundance of the mitochondrial tRNA^Leu(UUR)^ gene (determined by quantitative RT-PCR, forward primer: 5′-CACCCAAGAACAGGGTTTGT; reverse: 5′-TGGCCATGGGTATGTTGTTA) and with that of the nuclear β2-microglobulin gene (forward: 5′-TGCTGTCTCCATGTTTGATGTATCT; reverse: 5′-TCTCTGCTCCCCACCTCTAAGT).

### ATP measurement

Cellular ATP levels were measured with Luminescent ATP detection assay kit (Abcam) according to manufacturer’s protocol. The cells were cultured in the medium containing galactose instead of glucose and the readings were normalized to DNA abundance measured by Sybrsafe staining.

### Statistical methods

Group descriptive statistics are presented as mean ± SD and group count (percentage). The Kolmogorov-Smirnov test was used to test normality of the parameters (cord resistin, maternal Age, HbA1C, BMI, and gestational age). Among them, cord resistin, maternal age, and HbA1c were not normally distributed. Differences in characteristics between control and diabetic groups were assessed using Student’s t-test for normal distribution and nonparametric Mann-Whitney test for non-normal distribution. Maternal glucose (OGTT-2 h) and maternal factors which displayed significant difference between control and diabetic groups, including maternal age, HbA1C, BMI, and gestational age, were subjected to correlation analysis with cord resistin. Spearman correlations were used for correlation analysis for non-normal distributions. Multiple regression analysis was conducted to further assess relationships after controlling multiple variables. In the multiple regression model, cord resistin was the dependent variable and study groups (control and diabetes), maternal age, BMI and gestational age were the independent variables. The statistical analysis were performed in Excel, GraphPad Prism, and SPSS. For all analysis, *P*-values < 0.05 were treated as statistically significant.

## Results

### Cord blood resistin concentration is increased in offspring born to mothers with diabetes during pregnancy and correlates with maternal blood glucose levels

Demographics for participants providing cord blood samples are shown in Table 1. The participants with diabetes during pregnancy were older, had higher HBA1C, BMI, and slightly lower gestational age. There was no significant difference in the ethnicity and fetal sex between pregnant women with or without diabetes.

**Table 1:** Characteristics of research subjects providing cord blood samples

	DM *N* = 42 (Male 21; Female 21)	Control *N* = 81 (Male 37; Female 44)	***P***-value (DM vs Control)
Maternal Age, Y	31 ± 5.9	24.47 ± 4.4	*P* < 0.001
Maternal HbA1C, %	5.68 ± 0.74	5.15 ± 0.28	*P* < 0.001
Maternal BMI	32.74 ± 6.3	28.22 ± 6.77	*P* < 0.01
Gestational age, weeks	38.89 ± 0.66	39.55 ± 0.95	*P* < 0.001
Race			
Native American	24 (57%)	56 (69%)	*P* > 0.05
Hispanic	18 (43%)	25 (31%)	

**Fig. 1:**
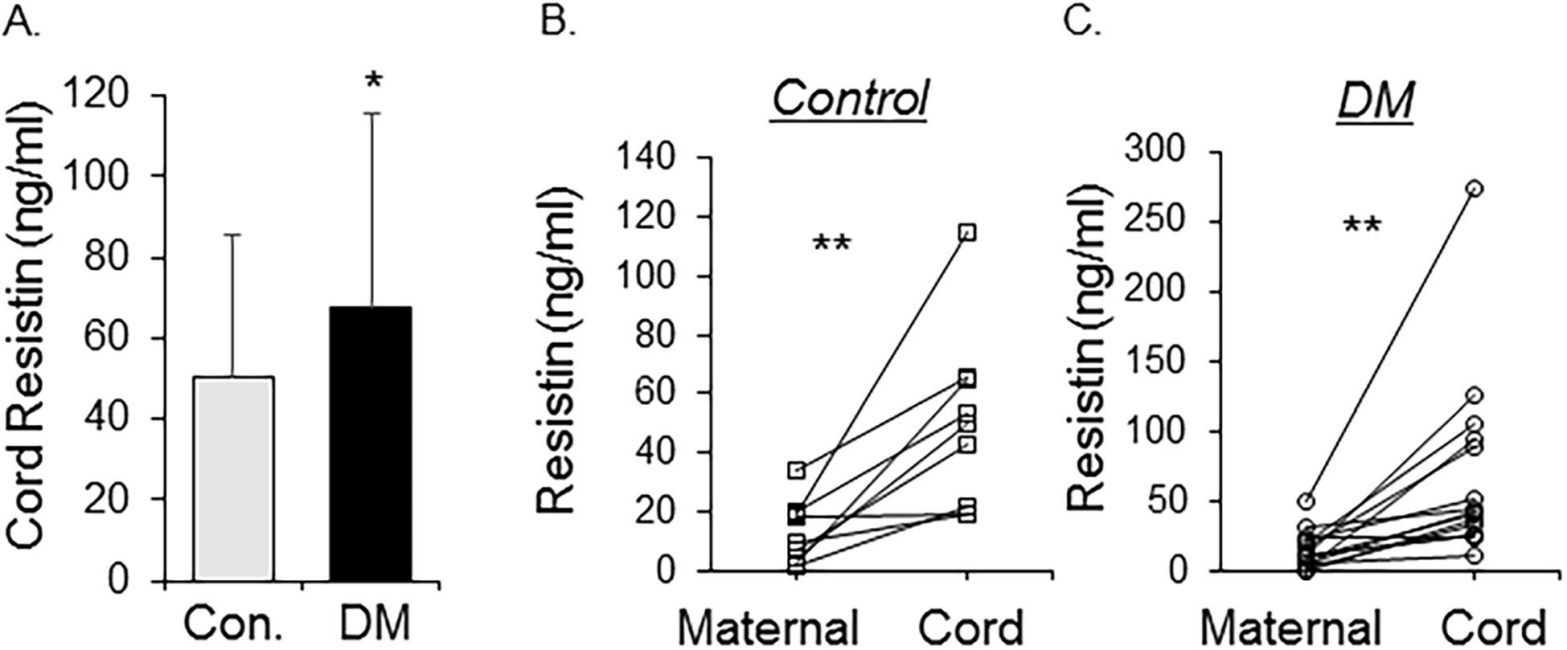
Serum resistin levels in cord and maternal blood. **a** Resistin concentrations were higher in the cord blood of diabetic women (DM) compared to healthy controls (Mean ± SD, * *P* < 0.05, *N* = 81 in control, *N* = 42 in DM); **b** and **c** Pairwise comparison of resistin levels in maternal and cord blood. *N* = 9 pairs of control (**b**) and *N* = 15 pairs of Diabetes (**c**), ** *P* < 0.01 between maternal and cord levels

**Fig. 2:**
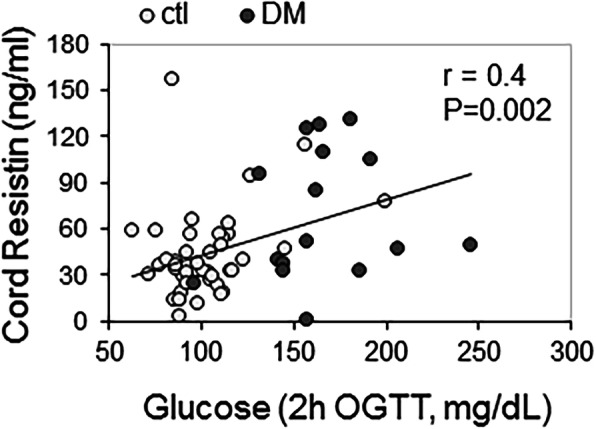
Correlation between cord blood human resistin and maternal blood glucose levels during pregnancy. Cord blood resistin levels were determined as described in methods. Maternal glucose concentration was measured at 2 h during the second trimester OGTT. *N* = 59 including 43 control mothers and 16 with diabetes

The level of resistin in cord blood of infants born to mothers with diabetes (67.3 ± 48.4 ng/ml, *n* = 42) were significantly higher (*P* = 0.03) than those born to control women (50.4 ± 35.2 ng/ml, *n* = 81) (Fig. 1a). Cord blood resistin levels were significantly higher compared to the corresponding maternal blood resistin levels in control (Fig. 1b) and women with diabetes (Fig. 1c). Concentrations of human resistin in cord sera correlated significantly (*R* = 0.4, *P* = 0.002) with maternal glucose concentrations measured 2 h after oral glucose challenge (OGTT, Fig. 2) during the second trimester of pregnancy. The correlation of cord blood resistin concentrations with maternal HbA1C at delivery was approaching significance (*P* = 0.057), whereas there were no significant correlations between cord blood resistin with maternal age, BMI, or gestational age at birth (Table 2).

**Table 2:** Spearman tests on correlation of maternal factors with cord blood resistin levels

	R (Correlation Coefficient with Cord resistin)	Correlation with Cord resistin
Maternal Age, Y	0.082	*P* > 0.1
Maternal HbA1C, %	0.207	*P* = 0.057
Maternal BMI	0.028	*P* > 0.1
Gestational age, weeks	0.059	*P* > 0.1

Multiple regression analysis was conducted to further examine the relationship between fetal resistin and multiple variables. As shown in Table 3, only the presence of maternal diabetes reached significance in the regression model, indicating maternal age, BMI, and gestational age did not contribute to the different resistin levels between control and diabetic groups observed here.

**Table 3:** Multiple regression analysis on the variables

Variable	Dependable Variable: human Cord resistin	
	Standardized Coefficients Beta	Significance (*P*)
Diabetes During Pregnancy	0.279	*P* = 0.03*
Maternal Age	0.035	*P* > 0.1
Maternal BMI	0.028	*P* > 0.1
Gestational age	0.059	*P* > 0.1

* *P* <  0.05 statistically significant

### Secretion of resistin from cord blood mononuclear cells in response to metabolic stresses

**Fig. 3:**
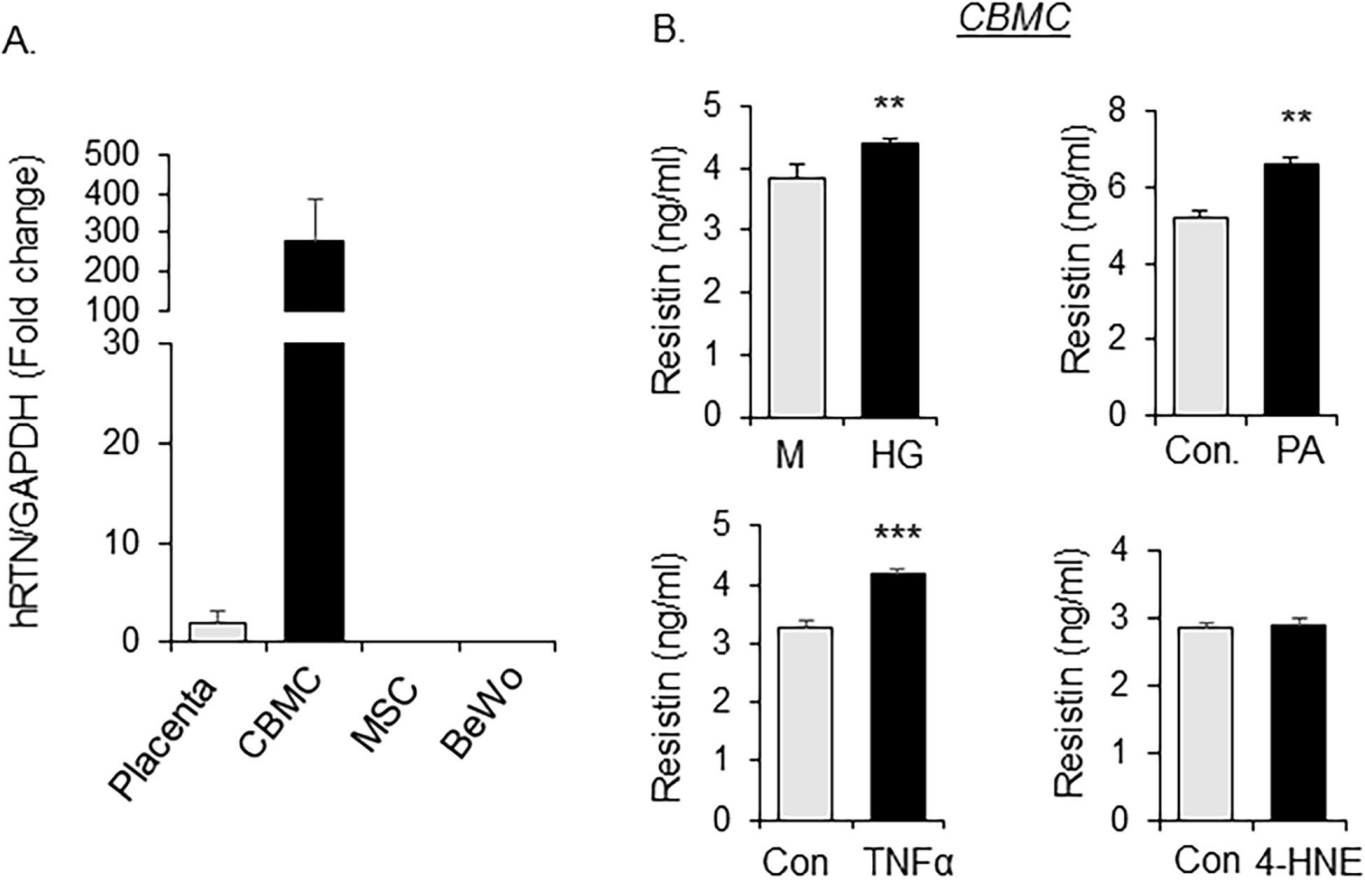
Secretion of resistin from cord blood mononuclear cells in response to metabolic stresses. **a** Expression of resistin mRNA in placenta and cord blood mononuclear cells (CBMCs), cord tissue mesenchymal stem cells (MSC) of healthy subjects (*N* = 5 subjects for placental tissues and CBMCs; *N* = 3 subjects for MSCs), and BeWo cells (trophoblast cell line) were measured by quantitative RT-PCR; **b** Mononuclear cells were isolated from cord blood of healthy pregnant women and were treated with TNFα (100 ng/ml), high glucose (25 mM), palmitate acid (0.6 mM), or 4-Hydroxynonenal (4-HNE, 0.6 mM) for 16 h, followed by measuring resistin levels in culture media with ELISA. M: mannitol treated group as an osmotic control for high group treatment. Mean ± SD, ** *P* < 0.01; *** *P* < 0.001; *N* = 4

The expression of resistin mRNA in fetal tissues and cells was examined by quantitative real-time PCR. As shown in Fig. 3a, resistin was highly expressed in cord blood mononuclear cells (CBMCs). Placenta also expressed resistin but at much lower abundance, whereas expression of resistin was not detectable in mesenchymal stem cells isolated from umbilical cord Wharton’s Jelly, nor in BeWo cells, a placental trophoblast cell line (Fig. 3a). Treating CBMCs with high glucose, palmitate, or the inflammatory factor TNFα, but not the oxidative stress inducer 4-HNE, resulted in increased levels of resistin in the culture media (Fig. 3b).

### Human resistin inhibits placental mitochondrial biogenesis

**Fig. 4:**
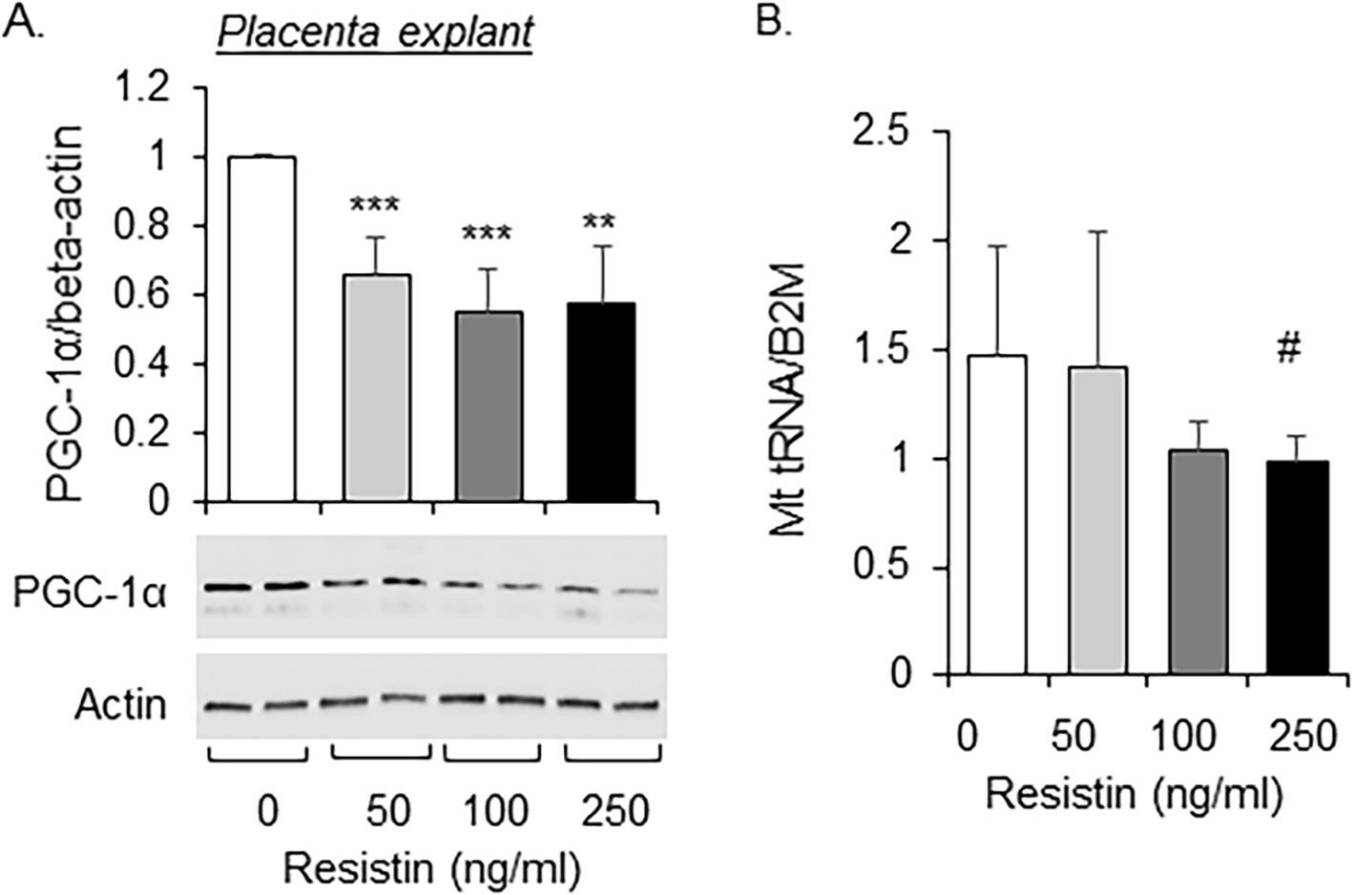
Effect of resistin on mitochondrial biogenesis in placentae. Human placental explants were treated with indicated doses of human resistin for 24 h. **a** protein lysates were subjected for Western blot analysis; **b** total DNA were extracted and mitochondrial DNA copy number was determined by fold change of mitochondrial tRNALeu^(UUR)^ gene DNA copy number normalized to nuclear β2 microglobin (B2M) with quantitative RT-PCR. Mean ± SD, ** *P* < 0.01; *** *P* < 0.001; *N* = 4. # *P* < 0.05 with one-tail T-test

We previously reported a decrease in the PGC-1α/TFAM mitochondrial biogenesis pathway in placenta of mothers with diabetes (Jiang et al. 2017). Treating human placental explants with resistin resulted in a maximal decrease in PGC-1α protein abundance at 100 ng/ml (Fig. 4a) accompanied by a significant decrease in mitochondrial DNA copy number (Fig. 4b), demonstrating the capacity of resistin to regulate placental mitochondrial biogenesis.

### Human resistin decreases PGC-1α and mitochondrial energy metabolism in placental trophoblasts

**Fig. 5:**
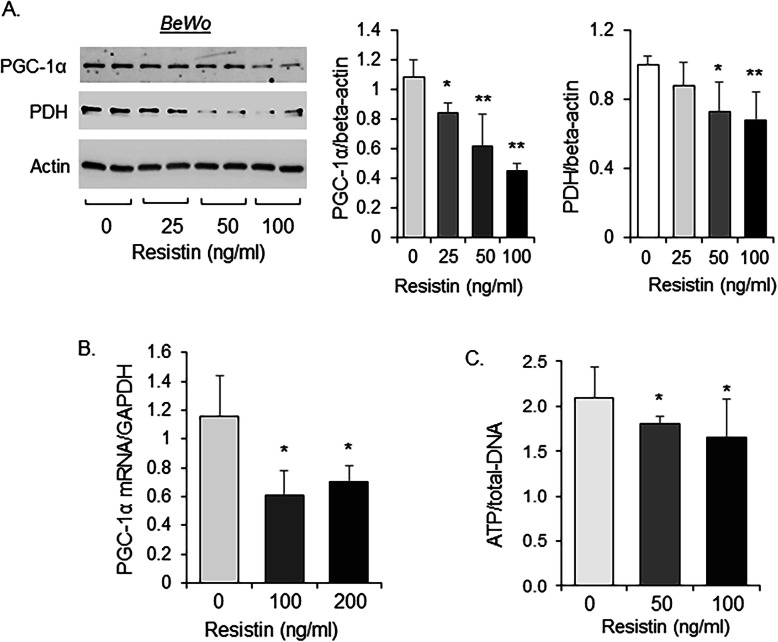
Effect of resistin on PGC-1α and mitochondrial metabolism in placental trophoblasts. BeWo cells were treated with indicated doses of human resistin for 24 h. **a** Protein lysates were subjected for Western blot analysis (*N* = 4 in each group); **b** total RNAs were reverse-transcribed and subjected for RT-PCR (*N* = 4 in each group); **c** BeWo cells were treated with indicated doses of human resistin in medium containing galactose for 24 h followed by assay for ATP levels (*N* = 8 in each group). Mean ± SD * *P* < 0.05; ** *P* < 0.01

Trophoblasts are the placental cells which provide the major source of nutrients for the growing embryos. In a transformed trophoblast cell line, BeWo cells, human resistin treatment also decreased the PGC-1α protein abundance (Fig. 5a) and its mRNA expression (Fig. 5b). In addition, the protein level of pyruvate dehydrogenase (PDH) was decreased by resistin treatment (Fig. 5a). Resistin also inhibited cellular ATP production (Fig. 5d), further demonstrating the influence of resistin on mitochondrial energy metabolism.

## Discussion

An adverse maternal environment, such as diabetes during pregnancy, impacts fetal and placental development, which is associated with increased risk of metabolic diseases in offspring later in life (Fetita et al. 2006; Dabelea and Crume 2011; Damm et al. 2016; Friedman 2018). The present study demonstrates that an increase in cord blood resistin found in the presence of maternal diabetes may play a role in placental mitochondrial biogenesis and function.

A recent meta-analysis of 18 published studies (Hu et al. 2019) notes that resistin levels are elevated in maternal circulation in gestational diabetes. Much less is known about the determinants of resistin abundance in the fetal circulation and current reports regarding the association between cord blood resistin with diabetes during pregnancy are discordant (Shang et al. 2018; Oncul et al. 2013; Mohamed et al. 2010). The present study found an increase in cord blood resistin in maternal diabetes, which agrees with the reports by Shang et al. and Oncul et al. (Shang et al. 2018; Oncul et al. 2013). We also demonstrated that resistin expression was highly enriched in cord blood mononuclear cells, suggesting that fetal mononuclear cells may be the main source of fetal circulating resistin. We, along with others (Chen et al. 2005; Erol et al. 2016), detect expression of resistin in the placenta. However, we found much lower abundance there compared to that in mononuclear cells. As no expression was detected in the placental trophoblast cell line, BeWo, we suspect that placental macrophages, rather than trophoblasts, are responsible for placental resistin expression.

In addition to cord blood resistin concentration being higher in pregnancies complicated by diabetes, a positive correlation with maternal blood glucose levels was found. Hyperglycemia and hyperinsulinemia in GDM are known to activate inflammatory cells and induce a pro-inflammatory status (Pantham et al. 2015). In accord, we found that cord blood mononuclear cells secreted resistin in response to exposure to high glucose and other diabetes-related factors. Thus, our findings further characterize resistin as an inflammatory cell-derived factor that responds to hyperglycemia and metabolic stresses associated with diabetic pregnancy. Our study begins to examine potential functions of resistin in the fetus, suggesting involvement in regulation of placental mitochondrial abundance and function. Mitochondria play a key role in placental function, and defects in placental mitochondrial function and content are associated with impaired placental energetics and increased oxidative stress, which lead to adverse pregnancy outcomes (Mando et al. 2018; Clemente et al. 2017; Bijnens et al. 2019). We previously demonstrated a decrease in PGC-1α/TFAM/mitochondrial biogenesis signaling in placenta of women with diabetes during pregnancy (Jiang et al. 2017). Here we observed decreases in PGC-1α expression and mitochondrial DNA copy number when human placental explants were exposed to resistin. In addition, resistin treatment reduced the abundance of pyruvate dehydrogenase (PDH) and ATP production. Pyruvate dehydrogenase is a mitochondrial enzyme that catalyzes pyruvate oxidation, linking glycolysis to the Krebs cycle for ATP generation to meet energy demands (Park et al. 2018). Mitochondria are the primary source of ATP needed for placental growth, nutrient transport, and hormone synthesis. Therefore, increased expression of resistin may contribute to impaired placental mitochondrial biogenesis and function, as well as offspring adverse outcomes in pregnancies complicated by diabetes.

The strengths of the present study are identification of resistin as a fetal factor derived from inflammatory cells that is affected by maternal diabetes and demonstration of a role for resistin in inhibiting placental mitochondrial metabolism. Limitations of the present study include the differences in baseline characteristics (including maternal age, maternal BMI, and gestational age) between the control and diabetic groups which could complicate data analysis and interpretation. However, to improve the assessment on their potential effects on resistin levels, these factors were statistically adjusted by the multiple regression analysis which suggested they were unlikely to contribute to the difference of resistin levels between control and diabetic groups. Also, the specific downstream signaling underlying resistin effects on mitochondrial biogenesis and the roles of resistin on other fetal tissues remain to be explored. Four distinct receptors have been identified to bind to resistin, including Toll-like receptor 4 (TLR4), decorin, receptor tyrosine kinase-like orphan receptor 1 (ROR1), and adenylyl cyclase-associated protein 1 (CAP1) (Zhao et al. 2019; Miao et al. 2018; Lee et al. 2014). Resistin/TLR4 has been shown to inhibit AMP activated kinase (AMPK) (Miao et al. 2018; Hardie 2007), an important regulator of mitochondrial biogenesis (Hardie 2007). TLR4 and AMPK inhibition can be the potential mechanism underlying resistin-induced decrease in placental mitochondrial metabolism. However, which of these receptors and the specific downstream pathways responsible for the effects of resistin on mitochondrial metabolism remains to be investigated. In addition, elevated fetal resistin can potentially impact energy metabolism and development of other fetal tissues, such as muscle, which remain to be studied.

As much remains to be learned about the role of resistin during fetal life, the clinical implications of these findings await future studies and definition. However, maternal diabetes has both immediate and long-term effects on the offspring which are tied to energy management and mitochondrial function. Thus, human resistin could be a potential therapeutic target or a diagnostic marker for the short-term and long-term adverse pregnancy outcomes of diabetes during pregnancy. Future studies to determine whether the observed changes in circulating resistin persist in the postnatal period and whether they relate to indices of metabolic dysregulation are needed and are best addressed by longitudinal studies of the children born to mothers with diabetes during pregnancy. Direct proof of the role of resistin in the perinatal period would require manipulation of resistin exposure to the fetus in vivo*.* Such studies are perhaps best performed in non-human primates, as the biology of resistin in rodents appears to differ significantly from that in humans.

## Conclusions

Here we provide evidence that human resistin is increased in fetal circulation of infants exposed to the diabetic milieu and impairs placental mitochondrial biogenesis signaling. Increased resistin is likely a response of monocytes/macrophages to hyperglycemia and metabolic stresses associated with diabetes during pregnancy. Thus, resistin acts as a circulating factor linking inflammation and energy metabolism during fetal life and it may contribute to impaired placental mitochondrial metabolism in maternal diabetes.

## Abbreviations

CBMCs: Cord blood mononuclear cells
PGC-1α: Peroxisome proliferator-activated receptor gamma coactivator 1-alpha
GDM: Gestational diabetes
4-HNE: 4-Hydroxynonenal
PDH: Pyruvate dehydrogenase
